# Hospital and Institutional News

**Published:** 1914-02-14

**Authors:** 


### HOSPITAL AND INSTITUTIONAL NEWS.

### NEW SCOTTISH LUNACY COMMISSIONERS.

Mr. John Carswell, F.F.P.S.G., L.R.C.P. Edin., has been appointed a Medical Commissioner in Lunacy for Scotland. A Glasgow man, who was a member of the Departmental Committee appointed to inquire into the operation of the Inebriates Acts, Scotland, and lecturer on mental diseases at Anderson's College Medical School, his posts include that of medical officer for mentally defective children, Glasgow School Board. He is the author of papers on "The Care and Education of Weak-minded and Imbecile Children in Relation to Pauper Lunacy," the "Legal Control of Inebriates," and, among others, "Dangerous Lunatics Charged with Crime." Under the provisions of the Mental Deficiency and Lunacy (Scotland) Act, 1913, the Secretary for Scotland has appointed Miss Kate Fraser, M.D., and Mr. James P. Sturrock, M.D., C.M., to be Deputy Commissioners of the General Board of Lunacy for Scotland, which, we may remind our readers, will be known after May 15 next as the General Board of Control for Scotland. Miss Fraser, who is at present medical officer to the Govan Parish School Board, graduated at Glasgow University, and has held appointments at the Royal Hospital for Sick Children, Glasgow, and at the Consumption Sanatorium for Scotland, Bridge of Weir. Among her publications is an account of the "Use of Binet-Simon Tests in Determining the Suitability of a Child for Admission to a Special School." Dr. James Prain Sturrock, the present medical superintendent of the Criminal Lunatic Department, Perth, is a graduate both of St. Andrews and Edinburgh Universities, and has held appointments at the Stirling District Mental Hospital and the Midlothian and Peebles Mental Hospital, and his numerous publications on aspects of psychiatric medicine include contributions on "The Mentally Deficient Criminal," "The Criminal Inebriate Woman," and "Modern Aspects of Eugenics."

### A RADIUM ADVENTURE AT LIVERPOOL.

The story of loss and eventual recovery of a tube of radium at the Royal Infirmary, Liverpool last week makes amusing as well as interesting reading. Two tubes containing respectively 30 milligrams and 50 milligrams of radium were recently presented to the institution, the larger of which was valued at rather more than £1,000. One of the patients had both fastened to his face, where they were left for the night, but in the morning the visit of the radiologist discovered one to be missing. *X*-rays, which were at once used to discover if the patient had swallowed the tube, showed that he had not done so, and a search was made among the sweepings of the ward floor. Fortunately, the Corporation dust-cart which was moving the rubbish had not left the institution, and Professor Wilberforce, the University physicist, who was summoned at the instance of the secretary, Mr. Francis Moore, was able to give a practical workaday test to his electroscope by applying it to the cart, with the result that the presence of radium was indicated. The next day, for the location of the radium was made towards evening, Professor Wilberforce again returned, and with the assistance of Dr. Holland, the radiologist of the infirmary, examined the bucketfuls, which the latter handed out. The twelfth of these proved to contain the missing tube of radium, which was returned to the safe keeping of the *x*-ray department. It is an amusing incident, this use of the *x*-rays and electroscope to find radium, and should impress the lay public with the "practical" value of the higher branches of University studies. Without the electroscope would the tube have been recovered? Mr. Moore deserves great praise for his prompt resource, which is likely to be remembered by every hospital that mislays this substance, which it is now almost impossible to buy.

### NEW SECRETARY OF BRISTOL GENERAL HOSPITAL.

We are informed that Mr. T. W. Gregg, at present secretary of the Belgrave Hospital for Children, has been appointed secretary of the Bristol General Hospital, in succession to Mr. William Thwaites. As there was a large number of candidates for this post Mr. Gregg is to be congratulated on his success; but it is one which will not surprise anyone who, like ourselves, is acquainted with his record. Not quite four years ago—to be precise, in June 1910—as The Hospital recorded at the time, Mr. Gregg was appointed secretary of the Belgrave Hospital. He entered institutional life at the age of sixteen by becoming junior clerk at the East London Hospital for Children; and when Mr. Thomas Hayes was succeeded by Mr. Wilcox he was promoted to the post of senior clerk, and then, in due course, became a secretary himself. The Belgrave Hospital for Children possesses forty beds, and the Bristol General 200, so that it is an important increase of responsibility which the new secretary undertakes. He is only thirty years of age, and arrives at his new post at a time when the work is likely to increase from the reconstruction schemes which are looming ahead.

### SHOULD HOSPITAL STREET COLLECTING BE STOPPED?

The recent success of certain flower days during the London season, when a vast amount of nominal selling proceeds on behalf of charity, has encouraged street collections generally, and the Charity Organisation Society met on Monday last to consider the rights and wrongs of this question. Mr. E. C. Price read a paper, in which he emphasised the growth of this practice, instanced the frauds to which it lent itself, and urged that no single charity should be allowed to collect in the streets more than one day in the year. This, he urged, would kill the "industry" now created, while safeguarding such time-honoured customs as

Hospital Saturday. Sir Edward Brabrook then proposed a resolution that street collecting tended to bring discredit on the cause of charity, which was carried, and which, we understand, is being circulated among the institutions, which we invite to send us their views on the subject. Lord Methuen, who presided, suggested that certificates should be given to all institutions and charities which desired to give proof of their *bona fides* should people whom they importuned ask them for a guarantee. Here are two suggestions, each worth debating. What are our readers' views?

### MEDICAL EVIDENCE IN THE LIVERPOOL MURDER CASE.

A curious feature was introduced into the Liverpool murder case last week, at which Ball, *alias* Sumner, was sentenced to death for the murder of Miss Bradfield, by an experiment which was described in the evidence of Dr. Broad. It will be remembered that the body of the murdered woman was tied up in a peculiar manner before being placed in the sack in which it was secreted, and Dr. Broad explained that his consulting room was the scene of an experiment in which the actions required in the tying process were carefully timed. He stated that the solicitor of Eltoft, who was implicated in the case and sentenced to four years' penal servitude as an accessory after the fact, accompanied by his counsel and a third person, called on him and in his presence tied up the third person with ropes and stitched him up in sacks in an identical manner with that in which Miss Bradfield's body was packed up. The time taken was four minutes and five seconds, and though all the materials were ready and there were obviously no "traces," such as blood, to be removed, the operation was performed by one man unassisted. The experiment was made in the interest of Eltoft, to show that his assistance was not necessary for the time in which the original act must have been done, and thereby to support his case of non-implication. Doctors' consulting rooms have witnessed many queer scenes before now, but this latest "experiment" is something in the nature of a new departure, and opens up a new function to the medical witness.

### THIRTY YEARS AS WORKHOUSE MEDICAL OFFICER.

When Mr. Samuel Craddock resigned the post of medical officer to the Bath Workhouse at the beginning of this month he had completed no fewer than thirty-one years' service, during which time it may be said he has seen the transition which has taken place between the complete neglect of skilled attention to the sick and modern infirmary methods. Himself the son of a medical man, and one of the founders of the Cottage Hospital at Shepton Mallet, to which he became medical officer, he took up the appointment which he has just resigned in 1883, when there were some 200 patients in the sick wards and no certified nurse to look after them. It was, in fact, the custom at Bath and elsewhere to employ what were known as "pauper helps" to tend the sick, and one of Mr. Craddock's early successes in administration was to secure the appointment of two trained nurses. This was not the only reform which he pressed upon the Board of Guardians, and by carefully forwarding to the Local Government Board a copy of every requisition which he made to the Board of Guardians, two inspectors eventually arrived, and directed the carrying out of his suggestions. He was anxious to procure special accommodation for consumptive cases, and when the scheme which he had discussed with the then chairman of the hospital committee was eventually adopted, it is recorded that no allusion at the opening was made to his share in this reform. This record of work is an interesting example of the fine part played by Poor-Law medical officers in raising the standard of the infirmaries during the past thirty years; perseverance, repeated efforts, good temper, and firmness here, as elsewhere, have been the agents in success. Mr. Craddock has also been Coroner for North Somerset for forty-seven years, the appointment at that date being made by the freeholders of the division, and the polling is said to have cost his predecessor £1,500. When the history of the Poor-Law infirmary comes to be written the story of such careers as this will form one of its most interesting parts. It is disappointing to note that the reason for Dr. Craddock's retirement is the new Poor-Law Order which comes into force on March 31, the day after his resignation is to take effect. He does not feel disposed to undertake the new clerical work which falls upon the medical officer.

### PIONEERS OF INFIRMARY REFORM.

In this connection a correspondent writes: "Dr. Craddock's resignation brings back to the memory of those interested in Poor-Law history the struggles of the pioneers of reform in the medical attendance and nursing of the sick in Poor-Law workhouses. The story of his long and strenuous fight on behalf of the sick poor is worthy of recall, for it has its lessons for to-day. Appointed in 1883, he found a workhouse containing sixteen sick wards with 230 beds, most of which were always occupied, and a nursing staff of one old woman suffering from heart disease and an untrained girl, assisted by pauper inmates. It was not, however, until 1894, as the result of his repeated remonstrances with the Guardians on the state of affairs, and the report of the Commissioner of the *British Medical Journal*, which was investigating at that time the condition of the sick wards of provincial workhouses, that the Local Government Board were induced to send one of their inspectors, Dr. Fuller, to hold an inquiry. The result of that inquiry was a complete confirmation of the position taken up by Dr. Craddock, and soon led to the introduction of radical changes. The following extracts from the reports of Dr. Fuller and the Commissioner of the *British Medical Journal* will give the workhouse medical officer of to-day an idea of what his predecessors had to contend against."

### INFIRMARY CONDITIONS THIRTY YEARS AGO.

"There was no hot water laid on in any of the wards—every drop had to be carried from a boiler across a court; and at night none was procurable except by boiling a kettle. Most of the wards were dark and squalid, and without any cross-ventilation. Dr. Craddock had managed to effect some slight improvement in the nursing staff, but for over 200 sick inmates, some sixty of whom were confined to bed, only one fully-trained nurse, assisted by two partly-trained women and a girl of seventeen, was available during the day. At night the patients were left entirely in the charge of paupers—two on the male side and one on the female. Dr. Fuller states in his report: 'As a natural sequence, at night, poulticing, when required, the care and cleanliness of the helpless and bedridden, and the administration of food and medicine to acute cases in stated quantities at stated times, are entirely neglected.' There were three roller towels, changed three times a week, for the use of each ward of about twenty-three beds; there was one comb 'somewhere about' for the patients, and 'perhaps a brush.' The bedding was chaff or flock, and the mattresses too thin to cushion the laths of the bedsteads. The medicines were left on the window-sills, and were administered by pauper inmates. In face of all these facts, a committee appointed by the Bath Guardians in June 1894, shortly before Dr. Fuller's inquiry, to investigate the complaints made by Dr. Craddock, reported: 'We cannot recommend that any addition be made to the present nursing staff of the infirmary.' They supported their conclusion by an argument dear to Guardians even to-day, that other infirmaries were in a worse plight still. Fortunately, Dr. Craddock was not the man to accept the Guardians' decision as final, and his triumph came a few months later, with Dr. Fuller's report, which initiated reforms which have continued until to-day."

### FARM COLONY FOR THE FEEBLE-MINDED OF SOMERSET.

As the result of an offer of over 100 acres of land in the Taunton district having been made by Mr. H. H. Wills, subject to the condition that some £14,000 be raised by the latter end of March, it is proposed to erect a farm colony for Somerset and Bristol for the permanent care of the feeble-minded, which will be modelled on the lines of such colonies as exist in Waverley in the United States. The scheme suggested provides for two houses, one for boys and one for girls, with the various necessary sick wards, schoolrooms, and manual-training rooms, to be increased by a future development of two other houses when the children attain the age of sixteen—one for men and one for women. It is the desire of the committee to secure permanent care of the feeble-minded, for which reason it is thought advisable to start with children of school age. When the necessary buildings and equipment have been obtained it is hoped that the cost of current expenditure will be met by payments of local authorities under the Mental Deficiency Act. There are some 500 feeble-minded children of school age in the districts named, and the scheme in the first instance is to provide accommodation for eighty to a hundred children.

### WIMBLEDON ISOLATION HOSPITAL: AN INQUIRY.

Owing to the complaints which have been received in regard to the control and administration of Wimbledon Isolation Hospital, the Public Health Committee of the Corporation have instructed the clerk to request the Local Government Board to hold an inquiry by one of its inspectors at the earliest possible date. The complaints, which appear to be concerned with cross-infection, include one from Dr. James Souter, who declared in a letter to the committee that, while his son was in the institution suffering from scarlet fever, he contracted diphtheria, while another resident in Wimbledon complains that his daughter, also under treatment for scarlet fever, contracted diphtheria in a similar way. Presuming the Board to accede to this request, the inquiry, whatever be its issue, will probably lead to a local recrudescence of opposition to the isolation hospital in general, on grounds with which cross-infection has now long made us familiar. The modern isolation hospital requires such skill in the administration and the nursing of its patients that the inquiry should be interesting to institutional workers of every class.

### FROM HOSPITAL SECRETARY TO PRESIDENT.

Mr. J. F. Hirst has been elected president of the Halifax Royal Infirmary in succession to the Rev. G. E. Aspinall. Mr. Hirst joined the board of the hospital in 1883, and for two successive periods has acted as honorary secretary. In 1889 the question of having a new institution to replace the old infirmary in Blackwall began with an inquiry, the report of which was approved, and so led to the building of the new infirmary at a cost of £100,000, during which process Mr. Hirst was one of the honorary secretaries. He then became a vice-president, and in turn auditor, a position which his new office has, of course, led him to relinquish. This remarkable record is further enhanced by the fact that every member of the present medical staff is believed to have been elected since he first sat at the board. Few hospital presidents, we imagine, can show a record which can equal this in the number of offices which they have previously held; but this promotion by active service is characteristic of the Royal Infirmary, Halifax, for the retiring president, to give merely one more example, was previously treasurer. The new president takes office at a time when the subscriptions are giving some anxiety, but as the reduction in the amount received from the workpeople's contributions is attributed to the Insurance Act, it will probably prove but temporary.

### A CHEESEPARING POLICY AT NORTH EVINGTON INFIRMARY.

We drew attention in these columns on January 24 to the medical arrangements at the North Evington Infirmary, Leicester, and pointed out that the proposal to appoint a resident medical superintendent at a salary of £400, rising to £450, and a visiting surgeon and physician at a salary of 52 guineas, was a cheeseparing policy which would, if unamended, seriously affect the efficiency of the work. That a cheeseparing policy does prevail and influence the Guardians disastrously at the present time appears from certain facts as to the staffing of the institution, which came to light at an inquest last week. The evidence disclosed the fact that one nurse is placed in charge of thirty patients, fourteen of whom are babies under the age of three years, and further, that these patients are distributed between three rooms, one of which is designated the nursery. Dr. Annie Fergus, the junior medical officer, explained that she had charge of the female side, women and children, that the number of nurses was in the hands of the matron and the committee, and that she had suggested in vain that the number of nurses should be increased. Dr. Fergus added that Dr. Grice, the senior resident medical officer, agreed with her, and was willing to appear.

### THE CORONER AND THE SPIRIT OF PARSIMONY.

The Coroner said that this statement of affairs was surprising, and that it would be thought that if the doctor recommended extra nurses they would be provided. The matron then explained that the staffing of the different wards was left to her and to the committee, and that there was no accommodation for more nurses. Taking the whole staff, the number of nurses was about one to ten patients. She would like to see it one to eight. We fully agree with the Coroner that lack of accommodation is no answer to the fact that there were too few nurses, and hope that the jury's rider, which specifically draws attention to the medical staff's opinion on this head, will awaken the Guardians to their grave responsibility in this matter. It is rather a curious commentary on their recent attempt to economise over medical salaries that they should also practise "economy" in their nursing staff, and hesitate to provide adequate quarters for them. The facts are worth placing side by side as illustrating a contention of three weeks ago, that work suffers all the way round once the spirit of parsimony is allowed to prevail in one quarter.

### A HOSPITAL AND A BULL-FIGHT.

We have had the opportunity of recording in these columns on various occasions both numerous and strange "by-ways" of raising money for institutional work, and a correspondent now informs us, on good authority, that recently at Teneriffe a bull-fight was organised and carried through for the financial benefit of the local hospital.

### RETIREMENT OF SIR LAURENCE GOMME.

Now that the London County Council, with its twenty committees, is responsible for so much work of a public health nature, any change in the officials becomes a matter of institutional interest. Thus the resignation of the Clerk, Sir Laurence Gomme, which was tendered at the Council's meeting on Tuesday, deserves record. Since he was appointed clerk in 1900, the working of the Midwives Act, and the preparation of a scheme for dealing with tuberculosis, to say nothing of the halting schemes by which London one day will be provided with an ambulance system under the Council, are all examples of the additional activities which have thrust themselves upon this body of recent years. In another connection, too, it is interesting to remind all Londoners that the naming of Kingsway and Aldwych were his suggestion, being, in fact, old names which he had discovered in the course of his topographical researches on an ancient map of the locality.

### AN ARCHITECT ON THE OPEN BASEMENT.

At a recent meeting of the Hampshire and Isle of Wight Association of Architects, which was held at the Royal Victoria and West Hants Hospital, Mr. G. A. Bligh Livesay, F.R.I.B.A., read an interesting paper on "A Provincial Hospital: its Design and Construction." After Mr. D. H. Robson Burrows, chairman of the hospital, had reminded those present that the two hospitals had amalgamated and were now working as one with three branches, and that the present building was also composite and in a transitory stage, Mr. Livesay detailed the architectural changes of late years. Mr. Keith Young is acting as joint architect for the administration block now rising, and he also assisted Mr. Livesay in designing the out-patient department, where the meeting was held. The main features emphasised by the speaker were the construction of the wards, which by "jacking up" the roof can be furnished with another storey, and further can be doubled in length so as to raise the existing number of sixty-eight beds to 178. The three large wards—for men, for women, and for children respectively—now contain eighteen beds apiece, while there are also three small wards of eight, four, and two beds. Coming to the design and its principles, Mr. Livesay pointed out that the hospital was built on the pavilion system, with the wards on the south and the administrative and kitchen blocks on the north side of the long main corridor. The part which interested Mr. Livesay most, however, may be given in his own words: "In section all the wards are built on open arches, so that the main floor is some six feet above the level of the ground, a form of construction which, though expensive, gives the undoubted advantages of raising the building to a purer stratum of fresh air, invites increased sunshine, and provides a continuous current of air underneath, as well as around and over the wards." On this point, and on the advantages or disadvantages of the sloping corridor, a discussion was invited by the speaker. The point is not an entirely architectural one, for the air space has a tendency to degenerate into a store-house for all kinds of rubbish. Such a thing ought not to happen, but it does, and so long as there is this risk the open-arch system is open to criticism. What are our readers' views?

### THE NEGLECT OF VOLUNTARY AID DETACHMENTS.

In a lecture delivered at Aldershot Dr. Balgarnie, Assistant County Director for Hampshire of the British Red Cross Society, sums up his views on the Voluntary Aid Detachments of the Territorial Force as follows. Voluntary aid is essential in time of war, and all civilised countries have recognised this fact. We are very many years behind foreign nations in this respect, having been the last to recognise the truth of Henri Dunant's views. We have now, however, a quite large enough body of men and women keen on the work and eager for improvement. Such enthusiasm should be freely encouraged by the authorities. At present the Voluntary Aid Detachments are wasted organisations. In time of war mobilisation would be next to impossible. There should be some high War Office official equivalent to the German Imperial Commissioner, who with a staff should devote the whole of his time to the matter of voluntary aid. The War Office might help enormously by allocating instructors to small groups, say, two or three County Divisions; and might also help by giving or lending such material as they could spare. We have many times alluded in these columns to the unsatisfactory state of the Voluntary Aid Detachments owing to official parsimony and neglect; so we are not surprised to find one who is in an even better position for judging confirming our own expressed views on this matter. The great difficulty is to stir the War Office into action. How can that be done?

### FINANCIAL CRISIS AT THE POLYCLINIC.

Those who remember the late Sir Jonathan Hutchinson with respect, and all who have benefited by the Medical Graduates' College and Polyclinic in Chenies Street, will be sorry to learn that it is in sudden financial straits. A sum, it seems, of £5,000 is immediately required; and as we understand that £2,000 has been promptly advanced by members of the profession who are interested in the institution, there is cause for believing that no reasonable foresight could have averted the present difficulty. The clinical demonstrations, laboratory work, and lectures carried on at the Polyclinic have made the institution an important teaching centre, at which medical men of different nationalities meet, and which is, for those who wish to keep abreast of modern medicine and the new specialties into which it is developing, a centre for the exchange of experiences and ideas. Mr. James Cantlie, 140 Harley Street, will gladly receive subscriptions in aid of the emergency fund.

### DEATH OF A HOSPITAL FOUNDER.

By the death of Mr. H. J. Casey, in his eightieth year, at Tunbridge Wells, the Leyton and Walthamstow General Hospital loses one, as a correspondent writes to remind us, who may be described as a founder, for in 1894, when it was realised that the then existent Cottage Home for Children at Walthamstow was no longer adequate for the town, Mr. Casey came forward and gave "Holmcroft," which the Council forthwith opened as a general hospital. Mr. Casey was a resident for many years in Walthamstow before going to reside at Tunbridge Wells, but on account of ill-health was unable to take an active interest in the hospital of which he was a trustee.

### THE BLACKPOOL HOSPITAL DEADLOCK.

A curious state of affairs appears to have arisen at Blackpool in connection with a dispute of some duration between the medical staff and the board of management of the Victoria Hospital. The trouble apparently arose through the conveyance of certain complaints to the committee by two nurses, who were subsequently dismissed. This led to a protest on the part of the honorary medical staff, who, if reports are to be believed, threatened to resign unless the two dismissed nurses were reinstated. The board let it be understood that they were making arrangements for carrying on the work of the institution, which has suffered a good deal in public estimation from the reports which have grown out of the dispute. It would seem fairly clear that there has been no adequate channel of communication between the medical staff and the committee of management, for this unwise division has often accounted for misunderstandings in hospital life. There must in fact be something radically wrong with the administration of any institution which allows matters to reach the stage which they appear to have reached here, and the committee would be well advised to restore public confidence by making a public explanation of the present position.

### THE PALLADIUM MATINEE.

The special matinée on behalf of the rebuilding fund of the Chelsea Hospital for Women, which will take place at the Palladium on March 17, in the presence of the King and Queen, is to be a worthy representation of all the arts and turns which go to make up a modern variety performance. Of the three hours which the performance is expected to last, two will consist of varieties and one of examples from what has been called facetiously the "monotonous" theatre, in the shape of two one-act plays. These are "How He Lied to Her Husband," by Mr. Bernard Shaw, and "A Social Success," by Mr. Max Beerbohm. With such a programme the special performance ought to outdo even the success which is the prerogative of such occasions.

### THE REGISTRATION OF NURSES.

An interesting discussion on this ancient controversial topic has been carried on during the past fortnight in the columns of the *Times*. Lady Helen Munro-Ferguson (wife of the new Governor-General of Australia) and Miss Haldane began it in a joint letter intended to promote the interests of the Registration of Nurses Bill, which has been annually before Parliament for ten years and seems to be making little headway towards the Statute-book. They used the arguments which have been familiar for many years in the mouths of those who favour this legislation; some, at least, of these arguments are worthy of attention, though it has always seemed to us that there is greater cogency in the reasons of those who oppose the registration of nurses. They were also sufficiently ill-advised to drag in the suffrage topic, which immediately played the part of a red-herring on the trail. This provoked Lady Jersey into a reply, in which she stated that Miss Nightingale was in favour of woman suffrage but an opponent of nurse registration. Lady Helen answered with a distortion of Miss Nightingale's views and work so grotesque that Sir E. T. Cook, whose admirable biography was reviewed in our columns last year (November 15, p. 165), felt obliged to write and correct the very gross errors of fact into which she had fallen. Then came a characteristic letter from Sir Victor Horsley, who began by describing Lady Jersey as in " mistaken opposition to the progress of civilised thought and political liberty." Of argument there was little in this letter, of assertion a great deal. It will be seen that the correspondence has thrown no new light on the subject under discussion, but has degenerated into hard words and misrepresentations. It is not thus that the numerous opponents of the Registration of Nurses Bill are likely to be converted.

### SALVARSAN WARD FOR THE LONDON HOSPITAL.

The Grocers' Company, two representatives of which sit on the board of the London Hospital, have given £10,000 for providing accommodation for the treatment of syphilis at the London. The out-patient committee recommended last November the establishment of a special department of this kind, but reported in favour of in-patient treatment. Most fortunately, too, it was determined to make no cruel attempt to discriminate between deserving or undeserving cases, infectivity being alone considered. The new quarters, which it is hoped will contain fifteen beds, will either be an addition to the Grocers' Wing of the institution or be placed in the isolation block. The President of the Local Government Board is known to favour the establishment of such a ward at the London Hospital.

### DEATH OF MISS JULIA COCK.

Miss Julia Anne Hornblower Cock, Dean of the London (Royal Free Hospital) School of Medicine for Women, has died at the age of fifty-four. Having qualified in 1882, she became an M.D. of Brussels in 1890. Formerly resident medical officer at Braintree and Bocking Cottage Hospital, and later senior physician to the New Hospital for Women, she became medical examiner to the Board of Education and of women proposers for Government Insurance. She also held the post of medical inspector of the North London Collegiate School for Girls and of the Camden School for Girls, and contributed the memorandum on " Medical Inspection of Secondary Schools for Girls " to the fifth volume of the Report of the Royal Commission on Secondary Education.

### INSTITUTIONAL PHARMACISTS AND THE PHARMACEUTICAL SOCIETY.

In a letter to the official organ of the Pharmaceutical Society Mr. Herbert Skinner, pharmacist to the Great Northern Central Hospital, raises the question why pharmacists in the public service should not band themselves together to put a representative on the council. He says that at present they are the only class not represented, and commends the idea to every institutional pharmacist. The suggestion is certainly deserving of consideration. There is among institutional pharmacists quite a number of men who have shown themselves to be capable of performing administrative duties of the kind which falls to the lot of members of the council of the Pharmaceutical Society. It should not be overlooked, however, that the present president of the Society, Mr. Edmund White, had a lengthy and distinguished experience of hospital pharmacy as pharmaceutist to St. Thomas's, and, although it is some years since he resigned his post, there are probably few men in pharmacy who understand the needs of institutional workers better than he. But still he is not a direct representative of institutional pharmacists, and Mr. Skinner's idea is undoubtedly a good one. Why should it not be carried out?

### THIS WEEK'S DRUG MARKET.

Business has been quieter this week, and the improvement noted last week has been barely maintained. The slight relapse, however, will, in all probability, be of a temporary character. Somewhat unexpectedly the price of santonin has again been advanced, this making the second advance this year; it is suggested that the further addition to the price of this drug is due to an increase in the amount of annual payment made by the syndicate controlling the Turkestan factory to the Russian Government for the monopoly of collecting the raw material from which santonin is produced. Opium is still practically unchanged. Essence of lemon continues to tend downwards in value. Indian hemp is dearer, and a further advance is considered not improbable. Castor oil has an upward tendency in price. Menthol is quiet, and the position is practically unchanged. Reports from the Norwegian fishing grounds are to the effect that stormy weather prevails which interferes with the fishing; the catch of cod and the yield of cod-liver oil are very much less than at the corresponding date last year; at present, however, it is too early to express an opinion of the future of the Cod-liver Oil market. At the recent public sale of drugs in Mincing Lane the demand was exceedingly quiet, and noteworthy changes in prices were few; honey tended lower, ipecacuanha was cheaper, and Jamaica sarsaparilla was dearer.

